# Predictive Factors for Outcomes of Selective Laser Trabeculoplasty

**DOI:** 10.1038/s41598-020-66473-0

**Published:** 2020-06-10

**Authors:** Matthew Hirabayashi, Vikram Ponnusamy, Jella An

**Affiliations:** 1 University of Missouri Columbia School of Medicine, Columbia, Missouri USA; 2 Mason Eye Institute, Department of Ophthalmology, University of Missouri, Columbia, Missouri USA

**Keywords:** Eye diseases, Glaucoma

## Abstract

We sought to  determine predictive factors for selective laser trabeculoplasty (SLT) outcome. 252 eyes from 198 adult patients with open-angle glaucoma who underwent SLT between July 2016 and February 2018 with a minimum 6 month follow up were reviewed. We defined success as ≥20% IOP reduction or ≥1 medication reduction without an IOP lowering procedure. We also evaluated the relationship of these factors to postoperative IOP elevation >5 mmHg (IOP spikes). Our primary outcome measure was association between age, type and severity of glaucoma, pigmentation of the trabecular meshwork (PTM), total energy delivered, and baseline intraocular pressure (IOP) with success. At 2 and 6 months, 33.6% (76/226) and 38.5% (97/252) of eyes met success criteria respectively. Baseline IOP > 18 mmHg was significantly associated with success both at 2 and 6 months, reducing IOP by 5.4 ± 5.3 mmHg (23.7% reduction), whereas those with lower baseline remained at −0.7 ± 4.6 mmHg (4.9% increase) at 6 months (*P* < 0.001). No other baseline characteristics significantly predicted success or IOP spikes. Patients with higher baseline IOPs had greater success rates and mean IOP reduction at both 2 and 6 months following SLT. Age, type and severity of glaucoma, PTM, or total energy delivery had no association with procedural success or IOP spikes. Patients with higher baseline IOP may experience greater lowering of IOP after SLT. However, SLT may be equally successful for patients with a variety of other characteristics.

## Introduction

Second in frequency only to cataracts, glaucoma causes more cases of blindness worldwide than any other ocular disease and currently affects over 70 million people, 10% of whom eventually lose their sight due to optic nerve damage^1^. Since it is estimated to continue increasing in incidence, refining treatment approaches become more crucial every year to preserve sight^2^.

Selective laser trabeculoplasty (SLT) reduces intraocular pressure (IOP) by improving aqueous outflow likely by biological rather than anatomic changes in the trabecular meshwork (TM) with proven efficacy ranging from 15–66% and a better safety profile than its predecessor, Argon Laser Trabeculoplasty (ALT)^1,3^. Originally indicated only for primary open-angle glaucoma (POAG), it has been shown to be efficacious for many other types of open-angle glaucoma^4^. Building on the principles of ALT, it uses frequency-doubled short pulse (Q-switched) Nd:YAG laser and selectively targets pigmented cells of the TM without damaging the overall anatomical structure of the meshwork^3^. The larger spot size of 400 μm for SLT compared to 50 μm for ALT allows for broader coverage of the angle and eliminates the need for as fine a focus while still providing the “champagne bubble” feedback that is generally recommended every two or three burns for appropriate energy delivery^5–7^.

Although efficacy and safety of SLT have been well documented, predictive factors for outcome of SLT tend to vary in the literature and large-scale investigations on this topic are limited. SLT has reportedly worked best for patients with ocular hypertension, POAG, pseudoexfoliation, or pigmentary glaucoma^7^. Pigmentation of TM (PTM) has not been associated with efficacy of the procedure^8^, but with higher risk of IOP spikes^9^. 360° treatment have been reported to be more effective than 180° treatment in one setting^10^, although many surgeons opt to treat 180° at a time in hopes of decreasing risk of IOP spikes.

With the growing interest in using SLT as first-line treatment and well known issues with medication compliance, determining which patients would have the best response to this therapy is increasingly important^11–13^. In this study, we attempted to determine possible predictive factors for both successful outcomes and common complications, including postoperative IOP spike following SLT treatment.

## Patients and Methods

We first obtained University of Missouri Institutional Review Board (IRB) approval who granted us a waiver of informed consent due to the retrospective nature of the study with deidentified data. All procedures performed in studies involving human participants were in accordance with the ethical standards of the institutional and national research committee and with the 1964 Declaration of Helsinki and its later amendments or comparable ethical standards. We performed a retrospective chart review of 252 eyes of 198 adult open-angle glaucoma patients who underwent SLT at University of Missouri between July 2016 and February 2018 in accordance with the guidelines and regulations of our IRB approval. All patients completed minimum 6 months follow up visit after SLT. We collected pre-op and post-op data at 2 and 6 month follow-up.

Our primary outcome measure was correlation between patient age, type and severity of glaucoma, PTM, or total energy delivery with procedural success. Success was defined as ≥20% IOP reduction or any reduction of medication without increasing IOP, and failure was not meeting these criteria or requiring an additional IOP-lowering surgery before the time point considered. Our secondary outcome measure was association of baseline characteristics to IOP reduction, medication reduction, and postoperative IOP spike (defined as elevation of IOP > 5 mmHg from baseline 1 hour after the procedure).

### Laser procedure and protocols

All SLT was performed with the 532 nm frequency doubled Q-switched Nd:YAG laser using 3 ns pulse and spot size of 400 micrometer. Power varied from 0.6 to 1.4 mJ and was titrated until microbubbles were visualized then reduced by 0.1 mJ. The settings for the SLT laser and therefore total energy varied depending on TM pigmentation. At 1 hour post procedure, a Goldmann applanation tonometer was used to check IOP by the treating physician or skilled ophthalmic technician. A drop of Timolol or Brimonidine was given to the operative eye prior to procedure, and no steroid was used postoperatively. No additional ocular hypotensive drops were given after the procedure unless the patient experienced an IOP spike. IOP was checked similarly at 2 weeks, 2 months, and 6 months post-procedure. Full slit lamp examination with gonioscopy and visual acuity testing were done at 2 weeks, 2 months, and 6 months post procedure. Patients remained on their medication regimen until 2 months where medications were adjusted at the physician’s discretion based on disease progression and individual IOP targets.

### Statistical analysis

We used paired *t*-tests to compare preoperative and postoperative IOP. A mixed model (age and preoperative IOP) and logistic regression (type and severity of glaucoma, pigmentation of the trabecular meshwork, total energy, and angle of treatment) with a random patient effect to account for individuals who contributed two eyes to the sample was used for determining predictive factors of success. Independent *t*-tests and Wilcoxon signed rank test to compare IOP and medication reduction based on preoperative IOP, a Mann-Whitney *U* test to compare total energy delivery between light and dense PTM groups, and independent *t*- and *chi-*square/Fisher’s exact tests to assess associations between factors and IOP spike. We reported all values as mean ± standard deviation for the data with normal distribution, median and interquartile range (IQR) for non-normally distributed data, and percentage (n) for categorical variables unless otherwise noted.

## Results

### Preoperative characteristics

Baseline characteristics are summarized in Table 1. The mean age of patients was 69.6 ± 11.2 years. Females comprised 49.2% of the sample size. Majority of patients were Caucasian (86.1%) with primary open-angle glaucoma (80.1%). The severity of glaucoma consisted of 43.3% (109/252) mild and 38.1% (96/252) severe according ICD-10 guidelines^14,15^. Mean preoperative IOP was 17.8 ± 4.4 mmHg and mean preoperative glaucoma medications was 2.0 ± 1.3 [2(2)].

**Table 1:** Baseline demographic and glaucoma status data (*n* = 252).

Age (years), mean ± SD	69.6 ± 11.2	Severity, n (%)	
Gender, n (%)		Mild	109 (43.3)
Female	124 (49.2)	Moderate	47 (18.7)
Male	128 (50.8)	Severe	96 (38.1)
Ethnicity, n (%)		Degree of angle treatment	
Caucasian	217 (86.1)	180°	174 (71.0)
Black	26 (10.3)	360°	71 (29.0)
Asian	6 (2.4)	Postoperative IOP Spike (>5 mmHg) n (%)	13 (5.1)
Hispanic	2 (0.7)		
Other	1 (0.4)	Baseline IOP (mmHg), mean ± SD	17.8 ± 4.4
Glaucoma type, n (%)			
Primary open-angle	206 (80.1)	Baseline medications, mean ± SD	2.0 ± 1.3
Normal-tension	31 (12.3)		
Secondary open-angle	15 (6.0)		

### Overall procedural success at 2 and 6 months

By our definition, 33.6% (76/226) of patients had successful SLT outcome at 2 months and 38.5% (97/252) at 6 months. Baseline IOP of 17.8 ± 4.4 (SD) mmHg was reduced to 16.7 ± 4.3 and 15.2 ± 4.9 mmHg at 2 and 6 months respectively, both statistically significant (*P* < 0.001). Baseline medications were reduced from 2.0 ± 1.3 [2(2)] to 1.6 ± 1.3 [2(2)] at 2 months and 2.0 ± 1.3 [2(2)] at 6 months. This change in medication number was not statistically significant at 2 months (*P* = 0.709) or 6 months (*P* = 0.578).

### Predictive factors for success

Findings for predictive factors for SLT success are reported in Table 2. The only baseline characteristic that significantly predicted success of SLT was baseline IOP at both 2 and 6 months (*P* < 0.001). Patients with baseline IOP of>18 mmHg experienced a mean IOP reduction of 3.7 ± 4.2 mmHg (17.3% reduction) at 2 months compared to −0.7 ± 4.1 (4.9% increase) mmHg in patients with baseline IOP ≤ 18 mmHg (*P* < 0.001). Similarly, at 6 months the patients with higher baseline IOP had mean IOP reduction of 5.4 ± 5.3 mmHg (23.7% reduction) compared to −0.7 ± 4.6 mmHg (4.9% increase) in those with lower baseline IOP (*P* < 0.001). IOP was not associated with mean medication reduction (*P* = 0.186).

**Table 2:** Predictive factors for SLT success at 2 and 6 months.

	Values		*p*-value	
	2 month	6 month	2 month	6 month
Age (years), mean ± SD			0.540	0.869
Success	70.8 ± 11.9	70.8 ± 11.5		
Failure	69.3 ± 10.3	69.2 ± 10.4		
Type (successful cases)			0.248	0.074
POAG, n (%)	63/76 (82.9)	79/97 (81.4)		
NTG, n (%)	9/76 (11.8)	13/97 (13.4)		
SOAG, n (%)	4/76 (5.3)	5/97 (5.2)		
Severity (successful cases)			0.588	0.311
Mild, n (%)	32/76 (42.1)	41/97 (42.3)		
Moderate, n (%)	14/76 (18.4)	18/97 (18.5)		
Severe, n (%)	30/76 (39.5)	38/97 (39.2)		
TM Pigmentation (successful cases)*			0.494	0.134
Light, n (%)	61/74 (82.4)	76/92 (82.6)		
Heavy, n (%)	13/74 (17.6)	16/92 (17.4)		
Total Energy Delivery (mJ), mean ± SD			0.325	0.989
Success	61.4 ± 26.7	64.2 ± 24.5		
Failure	62.1 ± 22.8	59.7 ± 24.2		
Baseline IOP (successful cases)			<0.001*	<0.001*
>18 mmHg, n (%)	56/76 (73.7)	75/97 (77.3)		
≤18 mmHg, n (%)	20/76 (26.3)	22/97 (22.7)		

*Light = trace – 2+ pigmentation, Heavy = 3 + – 4+.

### Predictors for IOP spikes

Our findings for the association between preoperative characteristics and IOP spikes are reported in Table 3. Overall, 5.1% (13/252) eyes experienced IOP spikes (defined as >5 mmHg elevation in IOP after laser surgery). IOP spikes was not associated with procedural success at 2 and 6 months, nor was it with age, type or severity of glaucoma, baseline IOP, PTM, or total energy delivery. None of the patients experienced any other adverse events related to SLT or vision loss ≥ 2 Snellen lines at any point during the follow-up.

**Table 3:** *P*-values for IOP Spike vs. patient characteristics or outcome measures.

Factor	*P*-Value
2 month success	0.342
6 month success	>0.999
Age	0.320
Type	0.650
Severity	0.668
PTM	0.740
Baseline IOP	0.400
Total Energy Delivery	0.627
Degree of Treatment	0.530

## Discussion

Many studies have attempted to characterize potential predictive factors of success following SLT and we sought to assess current claims and to contribute currently unexplored predictive factors such as total energy delivery during treatment. Our results agree with the frequently reported finding that baseline IOP is a predictor of SLT success^16–22^, while factors such as patient age, PTM, and type of glaucoma are not^17,20–24^. Some of the literature reports an association between heavily pigmented PTM and IOP spikes but we did not observe this in our population^9,24^. We found that baseline IOP significantly predicted success at both 2 and 6 months and those with higher IOPs (specifically >18 mmHg) had higher probabilities for a successful outcome and greater lowering of IOP. This result may be the result of flooring effect of any Schlemm’s canal based procedures, where IOP lowering cannot be achieved beyond the level of episcleral venous pressure^25–27^. Age, type and severity of glaucoma, PTM, or total energy delivery did not significantly predict a successful outcome at either 2 or 6 months in our population.

The existing controversy around this topic is likely multifactorial and elements such as varying technique and laser settings, success criteria, study power, and clinical protocols can all influence findings. Predictive factors for SLT success is an increasingly important topic due to growing current interest in using SLT as a first line therapy^11^. The recent randomized large-scale multi-center trial comparing SLT to eye drops for first-line therapy of glaucoma and ocular hypertension (LiGHT trial) demonstrated 95% of eyes at target IOP at 36 months with only 6 IOP spikes in their 770 patients^11,12^. These high rates of SLT success compared to ours and other studies may reflect how treatment-naïve eyes may have a favorable response to laser trabeculoplasty improving TM outflow facility, and prior treatment with topical aqueous suppressants may reduce the natural capacity of the patients’ TM and physiologic outflow, thereby limited response to laser trabeculoplasty^28^. It may also be due to the flexible patient-specific definition of success used in that trial compared to our singular success criteria. Our success rate was consistent with other existing literature that included medically treated glaucoma that generally report rates from 15–66%^29–32^.

We also attempted to characterize predictive factors for postoperative IOP spikes. Of the patients in our study 5.1% (13/252) experienced a postoperative IOP spike >5 mmHg over baseline within 1 hours of treatment. None of the predictive factors we evaluated was associated with IOP spikes. Our rate of IOP spikes is also similar to existing literature including medically-controlled glaucoma patients in which spike rates are often 10% or less^33–35^. We found no other complications from the laser procedure and no patients had loss of vision of 2 Snellen lines or more at any point during 6 month follow-up.

While our sample size was greater than many studies on this topic, we are limited by the retrospective nature and the potential for unobserved confounders. Glaucoma patients were not randomly selected and those receiving laser treatment may had certain glaucoma characteristics compared to those managed on medications alone or by incisional surgery. Our sample group may not be generalizable to non-Caucasian ethnicities or types of glaucoma other than primary open-angle. Our conclusions for predictive factors for postoperative IOP spikes must also be interpreted with care given the small incidence number. We also had 54 patients who received bilateral treatment and a “crossover” effect on the contralateral eye is known to potentially result in a significant IOP reduction^36,37^. While this confounder is not eliminated, it was accounted for in statistical analysis. Additional prospective and randomized studies with a more diversified set of patients will help determine if theses baseline characteristics have the same role in predicting success in patients regardless of ethnicity or type of glaucoma.

We would also like to address the treatment of 180° vs. 360° of the angle. There are multiple existing reports of greater angle treatment resulting in greater response^10,38,39^. Since our focus was on the factors that result in more favorable SLT outcome we considered degree of treatment to be one of the factors as it represents a variation in the technique. Due to this, considered it as a surrogate for degree of treatment rather than analyzing the groups separately. This also addresses a relevant question for 180° treatment: “Is more better?” since spot number and energy delivery also represent variation in technique. We decided presenting the data this way also can inform clinicians that more power and more spots may not necessarily result in greater response.

Despite these limitations, we have demonstrated that SLT is an effective method to lower IOP and medication burden with a low risk of complication that may have particular benefit for those with higher baseline IOP. Age, type or severity of glaucoma, PTM, and total energy delivery appear to not significantly predict success and patients with a wide variety of these factors will likely benefit equally.
